# Single-nucleus DNA sequencing reveals hidden somatic loss-of-heterozygosity in Cerebral Cavernous Malformations

**DOI:** 10.1038/s41467-023-42908-w

**Published:** 2023-11-02

**Authors:** Andrew K. Ressler, Daniel A. Snellings, Romuald Girard, Carol J. Gallione, Rhonda Lightle, Andrew S. Allen, Issam A. Awad, Douglas A. Marchuk

**Affiliations:** 1 Department of Molecular Genetics and Microbiology, Duke University School of Medicine, Durham, NC 27710 USA; 2 Neurovascular Surgery Program, Department of Neurological Surgery, The University of Chicago Medicine and Biological Sciences, Chicago, IL USA; 3 Department of Biostatistics and Bioinformatics, Duke University, Durham, NC 27710 USA

**Keywords:** Medical genetics, Genotype, Cardiovascular genetics, Cerebrovascular disorders

## Abstract

Cerebral Cavernous Malformations (CCMs) are vascular malformations of the central nervous system which can lead to moderate to severe neurological phenotypes in patients. A majority of CCM lesions are driven by a cancer-like three-hit mutational mechanism, including a somatic, activating mutation in the oncogene *PIK3CA*, as well as biallelic loss-of-function mutations in a CCM gene. However, standard sequencing approaches often fail to yield a full complement of pathogenic mutations in many CCMs. We suggest this reality reflects the limited sensitivity to identify low-frequency variants and the presence of mutations undetectable with bulk short-read sequencing. Here we report a single-nucleus DNA-sequencing approach that leverages the underlying biology of CCMs to identify lesions with somatic loss-of-heterozygosity, a class of such hidden mutations. We identify an alternative genetic mechanism for CCM pathogenesis and establish a method that can be repurposed to investigate the genetic underpinning of other disorders with multiple somatic mutations.

## Abstract (web summary)

Here the authors establish somatic loss-of-heterozygosity as a genetic underpinning of Cerebral Cavernous Malformations (CCMs): using single-nucleus DNA sequencing, they show homozygosity of chromosomes 7p and/or 7q leads to biallelic inactivation of CCM genes in resected lesions.

## Introduction

Knudson’s two-hit mutational hypothesis, in which biallelic inactivation is required for phenotypic change, emerged over 50 years ago and has since been confirmed in a large number of cancers^1,2^. More recently, the two-hit mechanism has been observed for a number of vascular malformation syndromes^3–7^. An elaboration on Knudson’s model emerged decades later, in which the two-hits (biallelic loss-of-function variants) in the tumor suppressor gene may require a third hit, pertaining to an activating variant in an oncogene, for tumorigenesis^8^. Critically, activating mutations in oncogenes have emerged in a wide variety of non-malignant disorders^9^. In cancerous and non-cancerous disorders with a three-hit (biallelic loss-of-function variants and an activating variant in an oncogene) mechanism, identifying a comprehensive list of somatic variants can be challenging due to limited sensitivity of sequencing methods and the diversity of genetic insults that may drive pathogenesis (e.g. single-nucleotide variants, structural variants, epigenetic events, etc.). But a complete mutation profile of the lesion (germline and somatic mutations) provides information on the underlying biology of the lesion, and in some instances may have relevance to disease progression and treatment, as genotypic differences will lead to phenotypic divergence.

One particular disease where genetic diagnoses including all three hits has been elusive for many samples is Cerebral Cavernous Malformations. Cerebral Cavernous Malformations (CCMs) are well-circumscribed vascular lesions in the central nervous system, that have been described as appearing mulberry-like^10^. Such lesions are found in ~0.2–0.9% of the population, with tremendous diversity in clinical presentation varying from asymptomatic to severe neurological phenotypes including seizures, movement disorders, focal headaches and stroke-like effects^11–14^. Around 20% of patients present with multiple lesions due to an inherited form of CCM, which is caused by a pathogenic variant disrupting the CCM signaling complex (CSC), comprised of three CCM proteins (KRIT1, CCM2 and PDCD10*)*^15,16^. However, the majority of patients typically have a solitary sporadic lesion, with either somatically-acquired loss-of-function variants in a CCM gene or a somatically-acquired, activating variant in *MAP3K3*, which is negatively regulated by the CSC^17^.

Importantly, activating variants in *PIK3CA* are commonly identified in CCM lesions and recent work has shown such activating variants are enriched in the same cells as either biallelic loss-of-function variants in a CCM gene or a monoallelic activating variant in *MAP3K3*^18,19^. When considering CCMs driven by loss-of-function variants in a CCM gene, animal models have long shown that lesion formation requires both biallelic loss of a CCM gene and an additional growth stimulus. In most mouse models, even with homozygous loss of a CCM gene, lesion formation is confined to the early postnatal stages of life, when angiogenesis is still occurring in the hindbrain, thereby providing a growth stimulus^20–22^. Alternatively, complete loss of p53 in combination with a germline variant in CCM also leads to lesion development in mice^23^. The combined data from animal studies suggest that complete loss of a CCM gene alone is insufficient to generate lesions. Thus, a preponderance of evidence from human lesions and from animal models suggests CCMs are generated from a combination of an activating variant in an oncogene such as *PIK3CA* or *AKT1*, which was recently identified in a single case^24^, plus either biallelic loss in a CCM gene or an activating variant in *MAP3K3*.

Even with the tremendous advancements in next generation sequencing and common use of high coverage targeted sequencing including genes known to cause CCMs, two critical facts remain true— (1) the majority of CCM lesions with reported DNA sequence analysis lack either an activating variant in an oncogene or either biallelic loss-of-function variants in a CCM gene or an activating variant in *MAP3K3* and (2) surgical resection remains the only available therapy for CCMs. Lack of a complete mutation profile, which we define for simplicity in this manuscript as having an activating oncogenic variant with either biallelic CCM loss-of-function variants or a monoallelic activating *MAP3K3* variant, may be driven by additional somatic variants in currently unidentified genes or variants in known disease-causing genes that are currently not being identified using existing sequencing strategies.

We hypothesized somatic loss-of-heterozygosity (LOH) in one of the three CCM genes may underlie a subset of hidden mutations. LOH is a genetic alteration where genetic information at a given chromosomal locus only includes a single parental chromosome and can be driven by a variety of mechanisms, including gene conversion, mitotic recombination or loss of part of or a whole chromosome. LOH frequently includes structural variants >1Kb in size and commonly encompasses multiple genes. Both copy number neutral (two alleles from a single parent) and copy number loss (a single allele) LOH in a subset of cells are mechanisms likely to be missed using targeted sequencing of bulk lesion DNA, given the size of events and inability to distinguish between somatic LOH and a single low-frequency variant. Critically, both sequencing and immunohistochemical approaches have found that somatic variants often occur in a small subset of cells, with some lesions showing evidence of <1% variant cells, which presents an additional challenge in identifying allelic imbalance, or somatic variants, in lesions lacking a complete mutation profile^18,19,25,26^.

In this work, we contend with such challenges by leveraging single-nucleus DNA-sequencing and the presence of somatic *PIK3CA* mutations in a fraction of the lesional cells to specifically establish somatic LOH as a mechanism for biallelic inactivation in CCMs. We show somatic LOH in three distinct patients and incidentally identify point mutations that yield the full complement of pathogenic variants in an additional three CCMs.

## Results

### Monoallelic variants commonly reported in CCM lesions

Recent work from our lab described the existence of genetic diagnoses of resected CCMs from 79 different patients and found a variant in either *MAP3K3* or a CCM gene in 46/79 CCMs. Further, in 15/32 lesions we identified one loss-of-function variant in a CCM gene^18^. Genetic analyses of other large cohorts of CCM lesions similarly report a significant number of lesions with monoallelic loss of a CCM gene or no known genetic cause^23,25^.

**Fig. 1:**
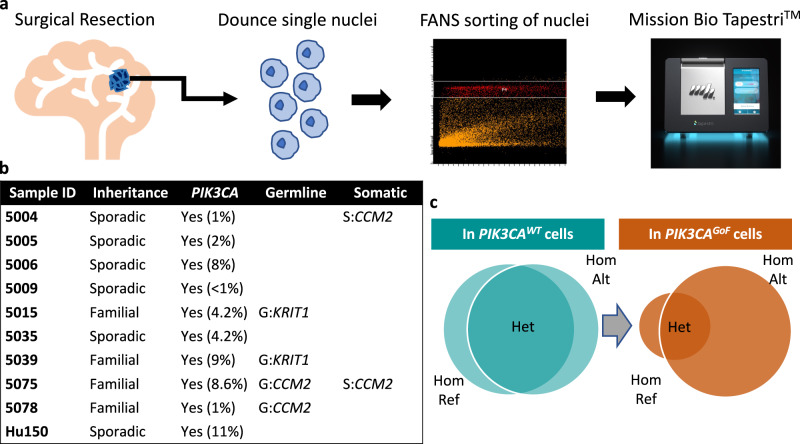
Identifying somatic loss-of-heterozygosity in resected CCMs. **a** Diagram of generation of single nucleus DNA-sequencing data. Frozen, surgically resected lesions are homogenized into single nuclei, sorted based on DAPI staining to enrich for high quality nuclei prep and sequenced using Mission Bio’s Tapestri^TM^ platform. **b** Panel of 9 lesions with an activating *PIK3CA* variant and either 0 or 1 loss-of-function variants in a CCM gene previously identified with targeted sequencing and/or ddPCR^26^. CCM 5075 is a control sample with a full complement of pathogenic variants, biallelic loss-of-function variants in a CCM gene (germline *CCM2* exon 2–10 deletion and somatic 1 bp deletion) and an activating variant in *PIK3CA*. S: Somatic, G: Germline. **c** Venn diagram representing the proportion of heterozygous calls that we expect to see in regions inclusive of a CCM gene in WT (teal circles) vs *PIK3CA*^*GoF*^ variant (orange circles).

We hypothesized that a subset of lesions lacking a complete mutation profile can be explained by large structural variants that are difficult to detect using common approaches that rely on targeted sequencing of bulk DNA. Specifically, we anticipated that somatic LOH underlies lesion genesis in a subset of CCMs with variants which were previously unidentified using existing diagnostic paradigms. To address this, we performed targeted single-nucleus DNA-sequencing (snDNA-seq) (Fig. 1a) with a sequencing panel that covered known genes implicated in CCMs, along with regions of significant variation on chromosomes 3,7,9 and 12. We analyzed 9 samples with a known *PIK3CA* variant and either 0 or 1 loss-of-function variants in a CCM gene and one sample with a complete mutation profile as a control lesion (Fig. 1b). We assessed whether the distribution of heterozygosity in common variants seen in dbSNP varied between cell populations with and without the known pathogenic variant in *PIK3CA* (Fig. 1c), leveraging knowledge that *PIK3CA* variants are co-mutated in the same cells that harbor variants in a CCM gene.

### No evidence of somatic loss-of-heterozygosity in *PIK3CA*^*GoF*^ cells in CCM lesion with biallelic loss of *CCM2*

We first assessed how the distribution of heterozygosity varied in a sample (CCM 5075) with a complete mutation profile – a germline deletion spanning exons 2–10 of *CCM2*, a somatic truncating single base deletion in *CCM2* and an activating variant in *PIK3CA*.

**Fig. 2:**
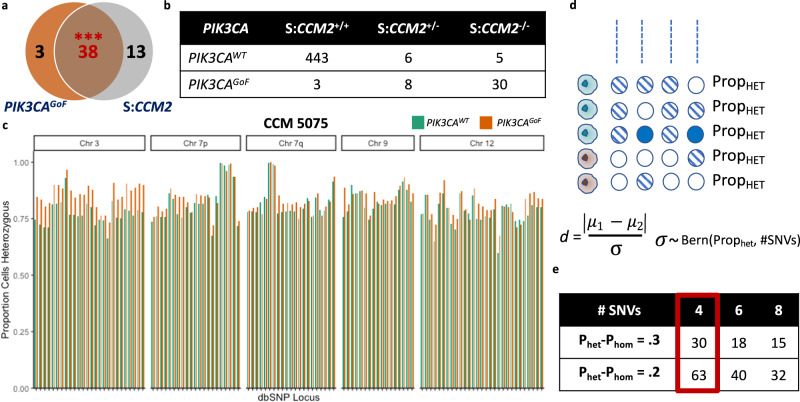
No evidence of loss-of-heterozygosity in a CCM lesion with biallelic loss-of-function variants in CCM2. **a** Significant co-enrichment of the sporadic *PIK3CA*^*GoF*^ variant and the somatic loss-of-function variant in *CCM2* (hg19 chr7:45112337:CA/C) in the same nuclei. **b** Critically, the somatic *CCM2* variant is within the region of the germline exon 2–10 deletion. Thus, these co-mutated cells are predominantly called as homozygous for the sporadic variant in *PIK3CA*^GoF^ cells (top row), while predominantly called as wild-type in *PIK3CA*^WT^ cells (bottom). Each column refers to number of nuclei genotyped as either wild-type (*CCM2*^+/+^), heterozygous (*CCM2*^+/−^) or homozygous (*CCM2*^−/−^) at the locus of the somatic 1 bp deletion. **c** The proportion of heterozygous cells at loci with common (reported in dbSNP) germline variants. Each set of teal and orange columns corresponds to the proportion of cells that are heterozygous for a given SNV. Exact chromosomal location and rsids for all loci in graph are reported in Supplementary Data 5. **d** Schematic representation and statistical model to quantify evidence of somatic loss-of-heterozygosity assumes all cells as independent Bernoulli variables with Prop_het_ defined as the proportion of SNVs in a given bin (bins of *n* = 4 shown) that are heterozygous (striped circles). A one-sided Student’s *t* test was used with delta being equal to the absolute value of the difference in Prop_het_ between WT and *PIK3CA*^GoF^ cells divided by the Bernoulli standard deviation. **e** Power analysis for different size bins performed and determined that as few as four SNPs in a bin is capable of identifying somatic loss-of-heterozygosity in 30 or 63 cells given a reduction in the proportion of heterozygous calls of 0.3 and 0.2 respectively (e.g. 80% of SNPs are heterozygous in WT cells versus 50% or 60% in variant cells). No set of 4 SNVs showed evidence of somatic LOH. ***p < 1e−4 using a two-tailed chi-squared test. Underlying data for bar charts included in source data.

snDNA-sequencing of CCM 5075 captures both the somatic single base deletion and the H1047R variant in *PIK3CA*. We see significant co-enrichment of both variants in sequenced nuclei, confirming that pathogenic cells harbor variants in both *PIK3CA* and *CCM2* (Fig. 2a). Importantly, evidence of the germline deletion is clearly apparent in pathogenic nuclei, as a significant majority (30/41) of *PIK3CA*^*GoF*^ cells were genotyped as homozygous at the locus of the identified somatic variant, which resides within the germline deletion (Fig. 2b). Biologically, we would not anticipate 8/41 cells to be heterozygous for the somatic variant, since the germline exon 2–10 deletion should lead to a single allele at that locus and either a wild-type or homozygous call. The presence of a non-trivial number of heterozygous cells illustrates the intrinsic variance in snDNA-seq data driven, in part, by technical artifacts due to nuclei doublets and especially, allelic drop-out^27^.

To address the issue of significant noise introduced by the aforementioned factors, we aimed to develop a statistical framework that would be robust to technical noise. Our model assumes that cells with and without activating variants in *PIK3CA* should be similarly confounded by technical artifacts and that distinct sequencing patterns between *PIK3CA*^GoF^ and *PIK3CA*^WT^ cells are driven by true biological differences. We specifically considered how frequently germline SNPs in single cells were genotyped as heterozygous versus homozygous for either the reference or alternate allele. Due to allelic dropout, most heterozygous sites across all covered regions tend to be called heterozygous in approximately 80% of nuclei (Fig. 2c), regardless of whether those cells do or do not have an activating *PIK3CA* variant.

We then determined whether or not variant cells (*PIK3CA*^*GoF*^) had a lower proportion of SNPs identified as heterozygous for a given predefined, genomic region (Fig. 2d). Such an approach allows for a certain level of technical artifact and for the potential that only a subset of variant cells may have somatic LOH, since the activating *PIK3CA* variant may proceed somatic LOH. A minimal genomic region definition was established by performing a power analysis to estimate the number of consecutive SNPs needed to theoretically identify LOH in a subset of CCM 5075 cells. We determined that 4 consecutive SNPs provided the requisite power given at least 30 variant cells (Fig. 2e). Importantly, we first established our statistical approach in a lesion with the full complement of variants already identified and thus expected no evidence of LOH across any of the 4 SNP bins. If we were to identify LOH in a given bin, that would likely suggest our approach may spuriously identify somatic LOH in certain situations. Promisingly, we see no evidence of somatic LOH across any 4 SNP bins or holistically in any chromosomal arm. Thus, we robustly assessed evidence of somatic LOH in a sample with a complete mutation profile and found clear evidence of a germline deletion leading to complete loss of CCM2 in *PIK3CA*^GoF^ cells, but no evidence of somatic allelic imbalance in any genomic region covered.

### snDNA-sequencing identifies somatic loss-of-heterozygosity in both sporadic and familial CCMs

We then extended our statistical approach to test for somatic LOH in *PIK3CA*^GoF^ cells in 9 CCM lesions, each from a different patient, with *PIK3CA* variants and either 0 or 1 deleterious variants in a CCM gene identified previously. We first assessed evidence of somatic LOH in any region on a given chromosome that encompassed four germline SNPs and found evidence of LOH in three samples across contiguous bins within chromosome 7. Critically, all three samples showed significant loss-of-heterozygosity for sets of four SNPs across either the entirety of chromosome 7 or for the entirety of one of the arms of chromosome 7.

**Fig. 3:**
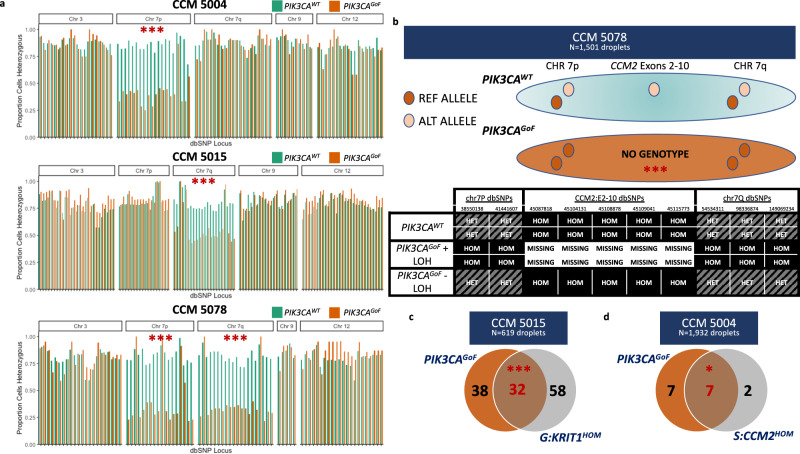
Somatic LOH identified in three resected CCM lesions using snDNA-sequencing. **a** In three out of 9 samples considered, we identified somatic LOH in cells with activating *PIK3CA* variants. All three identified the regions including the previously identified CCM variant. Each set of columns corresponds to the proportion of cells called as heterozygous for a germline SNV for cells with (orange) and without (teal) an activating variant in *PIK3CA*. Exact chromosomal location and rsids for all loci in graph are reported in Supplementary Data 5. **b** Top portion graphical representation of expected genotype when cells with a germline exon 2–10 deletion have an additional LOH event. Bottom shows in sample CCM 5078 all 5 germline dbSNP variants within the region of the germline *CCM2* exon 2–10 deletion had no genotype called (all 5 missing in 9/12 *PIK3CA* cells), consistent with complete loss of *CCM2* exons 2–10. Similarly, pathogenic variants in *KRIT1* and *CCM2* were enriched for homozygosity in *PIK3CA* variant cells in samples CCM 5015 (**c**) and CCM 5004 (**d**). ***p < 1e−4. *p < 0.01. *p*-values for **a** are generated using an unpaired, one-sided, ranked-sum Wilcoxon test that is Bonferonni corrected to account for multiple comparisons. Specific *p*-values are 2.28e−5 (CCM 5004 Chr7p), 1.38e−5 (CCM 5015 chr7q), 3.78e−8 and 7.8e−7 (CCM 5078 chrs7p and 7q, respectively). *p*-values for **b** determined using binomial test and *p*-value for **c** determined using two-tailed chi-squared test. Underlying data for bar charts included in source data.

Thus, we increased the size of bins considered and quantified the evidence of somatic LOH across all SNPs for all chromosomal arms and confirmed evidence of allelic imbalance in three out of nine lesions, with no evidence of loss in any other chromosomal arm within those samples, or in any other sample (Fig. 3a, Supplementary Fig. 1). Critically, all three regions of LOH encompassed a known, existing mutation in either *KRIT1* or *CCM2*. SNPs within the region of the germline exon 2–10 deletion of *CCM2* in CCM 5078 show evidence of complete loss of exons 2–10 in a subset of *PIK3CA*^GoF^ cells (Fig. 3b). Similarly, *PIK3CA*^*GoF*^ cells are enriched for pathogenic variants in *KRIT1* called as homozygous for sample CCM 5015 and in *CCM2* for sample CCM 5004.

Finally, we confirmed that the somatic LOH was not driven by unbiased allelic drop-out by confirming that common variants were predominantly called as homozygous for the same parental allele; the region of LOH consisted of predominantly a single haplotype and not random parental alleles (Supplementary Fig. 2).

### snDNA-sequencing identifies complete mutation profile in 6/9 samples with hidden mutations

Single-nucleus DNA-sequencing further allowed for the identification of additional pathogenic variants that were previously intractable but were not driven by somatic LOH. Specifically, we identified the *MAP3K3*^I441M^ variant in CCM 5006, as well as one and two deleterious variants in *KRIT1* in samples CCM 5039 and CCM 5009, respectively. Importantly, all identified variants were significantly enriched in *PIK3CA*^GoF^ cells (Supplementary Fig. 3b). Thus, we were able to provide a complete mutation profile for 6/9 lesions chosen for snDNA-sequencing (Supplementary Fig. 3a).

CCM 5009 is a particularly interesting case, elucidating an additional mechanism outside of somatic LOH that underlies a subset of hidden mutations. To validate the existence of pathogenic variants identified using snDNA-sequencing, we sampled two independent, separate regions of the lesion, and performed bulk targeted sequencing independently on each section (Supplementary Fig. 4a). Interestingly, one portion of the lesion sample led to validation of all three variants seen in snDNA-sequencing with minor allele frequencies between 1.8–3.3%, while the other portion did not exceed 0.2% variant reads for any of the three variants, suggesting that significant intralesional heterogeneity may impact the ability to identify pathogenic variants in CCMs.

### 100X whole genome sequencing similarly identifies allelic imbalance in CCM 5078, but not CCM 5004

**Fig. 4:**
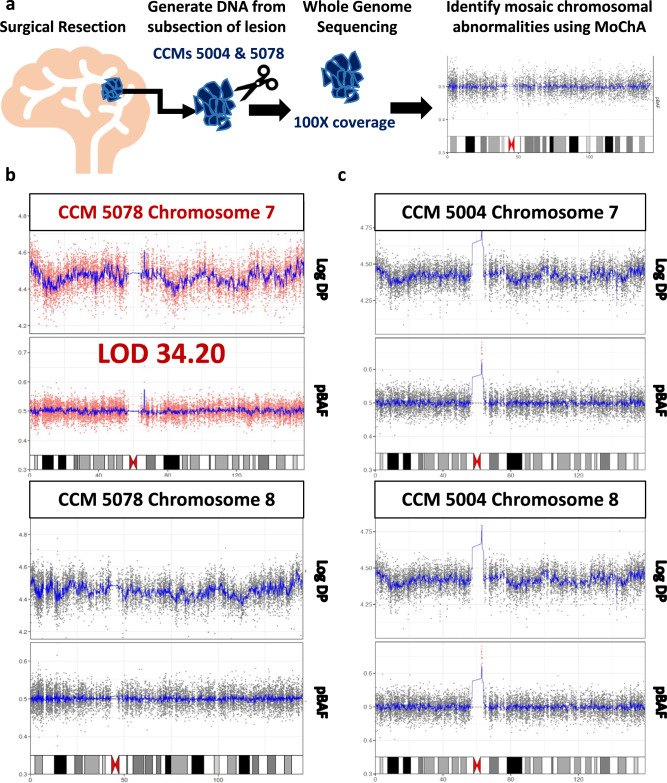
High coverage whole genome sequencing confirms allelic imbalance in chromosome 7 for sample CCM 5078. **a** DNA was extracted from subsections of lesions CCM 5004 and CCM 5078 and 100X WGS was performed prior to downstream analyses with MoChA. **b** Allelic imbalance in a small number of cells (~1.8%) was identified across the entirety of chromosome 7 in sample CCM 5078. MoChA results for chromosome 8 included below for visual comparison. Each dot represents either the log depth or predicted B-allele frequency for a given SNP. Red represents a region called by MoChA as having allelic imbalance. **c** Alternatively, MoChA was unable to confidently identify allelic imbalance in chr7p in CCM 5004.

We developed a single-nucleus DNA-sequencing based paradigm to identify somatic LOH, hypothesizing that LOH in single cells may be easier to identify than allelic imbalance in a small percentage of cells in bulk tissue. Recently, Loh et al. developed the MoChA software (https://software.broadinstitute.org/software/mocha/) to identify mosaic chromosomal alterations in as little as 1% of cells from bulk DNA^28,29^. To determine whether somatic allelic imbalance could similarly be detected using bulk DNA, we performed high coverage (100X) whole genome sequencing on the two lesions where we had access to additional high quality lesional DNA, CCMs 5004 and 5078 (Fig. 4a). We were able to identify allelic imbalance across the entirety of chromosome 7 in CCM 5078 (Fig. 4b). However, CCM 5004 showed no evidence of allelic imbalance using MoChA (Fig. 4c, Supplementary Data 1), which is likely due to a small fraction of variant cells. While whole genome sequencing cannot be used to determine whether or not allelic imbalance encompassing CCM genes occurs in the same population as activating *PIK3CA* variants, orthogonal validation of allelic imbalance in CCM 5078 suggests MoChA is capable of identifying allelic imbalance in CCMs when the region of loss encompasses large enough regions, such as an entire chromosome in this instance, or is in a large enough number of cells.

## Discussion

The genetic etiology of cerebral cavernous malformations has been significantly elucidated in the past decade due to the development of next generation sequencing and focused deep sequencing of CCM and CCM-interacting genes. Nevertheless, a significant number of CCM lesions with reported genetic diagnoses lack a complete mutation profile and harbor unidentified genomic variation. Within this work, we have taken lesions that previously contained such missing mutations and, using snDNA-sequencing, provided a complete mutation profile for 6/9 CCMs. Critically, in three of these samples, including both familial and sporadic lesions, we have identified an alternative genetic mechanism leading to biallelic loss of a CCM gene, somatic LOH.

Currently, the only existing treatment for damaging CCMs is surgical intervention. For patients harboring a germline mutation, lesions emerge throughout the brain and there is no approved treatment to either shrink lesions or prevent lesions from causing neurological dysfunction. A complete mutation profile alone will not immediately lead to improved patient outcomes; however, it may be a necessary first step towards determining how certain genotypes impact lesion aggressivity or response to emergent therapies.

We have shown that large structural variants in chromosome 7 exist in pathogenic cells within CCM lesions. While we have not distinguished between copy neutral and copy loss variation, both perturbations are likely to have phenotypic impact either on lesion growth or the propensity for hemorrhage, given the large number of genes encompassed within all three somatic LOH events identified. We note that we have not seen evidence of significant reduction in depth of coverage in regions of somatic LOH in CCMs 5004, 5015 and 5078 (Supplementary Data 2), which is consistent with copy neutral mechanisms. However, we cannot rule out copy loss due to the variability in read depth across nuclei and loci.

While uniparental disomy will not lead to phenotypic anomalies as frequently as copy number loss, a number of disorders are driven by such a genetic perturbation. In chromosome 7 alone, where we have identified somatic LOH in CCMs, maternal uniparental disomy has been found to drive CNS disorders such as argininosuccinic aciduria^30^ and Silver-Russell syndrome^31^. Similarly, copy loss of genes within chromosome 7 will cause a large variety of neurological and oncological disorders^32^.

While germline LOH events are clearly pathogenic, somatic LOH events will only impact a subset of cells. Importantly, somatic LOH of chromosome 7 has already been reported in the progression from myeloid dysplastic syndrome to acute myeloid leukemia, leading to a more severe presentation of disease^33^. Thus, we posit that the relationship between somatic LOH and disease progression is worthy of continued investigation, as such a mechanism may lead to distinct lesional characteristics compared to lesions with biallelic loss of a CCM gene driven by variants that do not extend into neighboring genes. Further, any of the large number of disorders that include an activating variant in an oncogene alongside biallelic loss of function variants can use the snDNA-sequencing based approach described above to better understand genetic underpinnings of disease.

Importantly, there remain significant obstacles in transitioning from the proof-of-principle findings within this work to impacting patient outcomes. A larger number of lesions with somatic LOH, alongside robust phenotypic data, would be an essential first step. Prioritizing samples with an activating *PIK3CA* and/or monoallelic deleterious CCM variants for snDNA-sequencing may accelerate discovery of somatic LOH events. Further, improvements in isolating variant cells prior to sequencing may allow for the identification of allelic imbalance using SNP- arrays on bulk samples.

An additional limitation of our approach to identify somatic LOH is the reliance on a previously identified activating variant in an oncogene, *PIK3CA*, and our approach is not applicable in samples without such a variant. Further, the variants in *PIK3CA* occurred on a different chromosome than the regions of LOH. Critically, allelic drop-out is a common artifact in single-cell and single-nucleus sequencing which can lead to correlated homozygous calls across a chromosome and may make identification of somatic LOH encompassing *PDCD10*, which is on the same chromosome as *PIK3CA*, intractable using our analytical approach.

Somatic LOH will only explain a subset of lesions lacking a complete mutation profile and we have not attempted to identify further genetic perturbations that may cause CCM lesions. Variants in CCM genes or *MAP3K3* may go unidentified for a variety of reasons other than LOH, including intralesional heterogeneity, as made apparent in the case of CCM 5009 (Supplementary Fig. 4). Critically, disease-causing genes yet to be firmly established as causal for CCMs may be responsible for a subset of lesions. As several signaling pathways are perturbed by loss of function of the CCM-signaling complex, or gain-of-function variants in *MAP3K3* and *PIK3CA*, additional genes may underlie CCM formation. Thus, a variety of sequencing strategies should be used to uncover more hidden mutations, including panels sequencing a diverse array of genes at high depth, as made clear by the existence of lesions within our CCM sample set^18^ without a complete mutation profile.

## Methods

### Ethical statement

Our research complies with all relevant ethical regulations, including the Declaration of Helsinki and has been approved by the Institutional Review Boards of University of Chicago, Duke University and the Alliance to Cure Cavernous Malformations.

### Cerebral cavernous malformation lesions

All human CCM tissue specimens have been previously reported^18,19^ and remaining portions of lesions stored at −80 °C in Tissue Tek^TM^ O.C.T. (Optimal Cutting Temperature) blocks were used for all newly generated sequencing data. All specimens were obtained from surgically resected specimens from one of the following: the Barrow Neurological Institute, Angioma Alliance biobank, or the University of Chicago.

### DNA extraction

DNA from human CCM samples was extracted using Qiagen’s DNeasy Blood and Tissue kit per the manufacturers’ directions. Final DNA concentrations were quantified per manufacturer’s instructions using Qubit dsDNA BR assay kit (Invitrogen cat. Q32850).

### Whole genome sequencing

For two of the three samples (CCMs 5004 and 5078) with identified somatic loss-of-heterozygosity we had sufficient tissue to extract DNA and perform 100X whole genome sequencing. Extracted DNA was sent over dry ice to Novogene and 100X whole genome sequencing was performed using the Novaseq PE150 with 350bp whole genome library preparation.

Following sequencing, germline single-nucleotide variants were similarly generated by Novogene using their standard analysis pipeline. Briefly, alignment to hg38 reference was done using BWA and germline SNP variants were called using GATK, both using GATK best practices parameters. Variants were then filtered using the below parameters:

QD < 2.0, FS > 60.0, MQ < 40.0, HaplotypeScore > 13.0, MappingQualityRankSum < −12.5, ReadPosRankSum < −8.0.

### Targeted DNA-sequencing

For all samples, targeted DNA-sequencing and variant identification was done as previously described^18,19^. For CCM 5009, additional targeted sequencing was performed. We generated targeted DNA-sequencing data from two separate locations of a remaining piece of frozen lesion with sequencing libraries prepared using the SureSelect XT HS target enrichment workflow (Agilent), consistent with previously reported targeted DNA-sequencing. However, we used a targeting panel with amplicons covering 14 genes, including all three CCM genes (*KRIT1*, *CCM2* and *PDCD10*), *PIK3CA* and *MAP3K3*, while the previously reported targeted sequencing used a panel with the following genes:

KRIT1,CCM2,PDCD10,PIK3CA,PTEN,AKT1,KRAS,RAF,NRAS,MAP2K1,RASA1,TEK,GNAQ,GNA11,MAP2K2,PPP2R5D,ACVRL1,ENG,SMAD4,AKT2,AKT3,CCBE1,CDKN1C,FLT1,FLT4,FOXC2,GATA2,GDF2,GJC2,GLMN,KIF11,MTOR,PIK3R2,PTPN14,SOX18,STAMBP,VEGFC,MAP2K4,MAP3K1,MAPK1,JAK1,JAK2,JAK3,KDR,NOTCH1,PDGFRA,PDGFRB,RET,HRAS,TP53,MSH2,MYB,MYCN,MYC,ERBB2,EGFR,NTRK2,ODC1,SLC25A21,PTTG1,TSC1,TSC2,EPHB2,TGFBR1,TGFBR2,TGFBR3.

All coding exons were covered for the three CCM genes, whereas only the exon(s) encompassing the known somatic mutation hotspots were covered for *PIK3CA* and *MAP3K3*.

After library preparation of the two samples from the same lesion, paired-end 150bp reads were sequenced across two lanes of an Illumina iSeq 100 sequencing system. Sequencing data was processed according to the GATK (Broad Institute) best practices for short variant discovery with tumor-only data. Processed sorted BAM files were viewed using IGV and read counts are displayed for variant loci in Supplementary Fig. 4.

### Single-nucleus DNA sequencing

Surgically resected frozen human CCM lesion tissue was prepared for snDNA-sequencing using a protocol originally described by L. Martelotto^34^. Briefly, frozen tissue was separated into single nuclei by Dounce homogenization in Nuclei EZ Lysis Buffer (Sigma-Aldrich), followed by filtration through 70 um and 40 um mesh. Once nuclei have been isolated and filtered, DAPI was added to the homogenate and nuclei were sorted using a FACSAriaII (BD) (70 um nozzle, 70psi, 4-Way Purity, chiller) gating to retain singlet DAPI-positive events^18,19^.

Sorted nuclei (≥75,000) were pelleted and resuspended in 30–50 uls of MissionBio Cell Buffer. Samples with <5% aggregate nuclei and 2000–4000 nuclei/ul after appropriate dilution were used for snDNA-sequencing. Library preparation was performed using Mission Bio’s Tapestri platform according to manufacturer’s protocols.

Libraries were generated with a custom amplicon panel (Supplementary Data 3) aimed at identifying a large number of germline variants (SNPs) with increasing density of coverage around known pathogenic genes, *MAP3K3, KRIT1, CCM2, PDCD10* and *PIK3CA*. While nearly all exons were covered for *KRIT1, CCM2* and *PDCD10*, hotspot regions were covered for *MAP3K3* and *PIK3CA* and other genomic regions were chosen due to being highly heterozygous. Libraries were sequenced with a NextSeq Mid-Output 2*150bp kit (Illumina) and data processing and QC was performed using MissionBio’s analysis pipeline.

### Analysis of single-nucleus DNA sequencing to identify somatic LOH

For all column graphs (Figs. 2, 3 and Supplementary Figs. 1 and 2) and quantification of somatic LOH at chromosomal arm level or across 4 SNPs, variants were processed as follows. MissionBio’s mosaic script was used to extract relevant variants from h5 files generated using MissionBio’s base analysis pipeline with alignment to hg19. Variants were filtered using mosaic according to the following parameters – minimum depth = 10, minimum genotype quality = 30, variant allele frequency reference = 5, variant allele frequency homozygous = 95 and variant allele frequency heterozygous = 15, minimum percent cells = 75 and minimum mutant percent cells = 50. Activating *PIK3CA* variants were manually inputted and whitelisted, since somatic variants would not otherwise pass these filters meant to extract germline variants.

Following initial filtration, variants were annotated with dbSNP ids and any variant not found in dbSNP was excluded. Variants were then exported into csv format and graphs were generated using excel. For graphs, variants were limited to single-nucleotide changes to limit potential alignment and call biases that may occur with indels and all graphs (excluding Supplementary Fig. 2) show proportion of cells called as heterozygous given the above filters.

For power analysis of LOH in CCM 5075 (Fig. 2), we performed a one-sided Student’s *t* test with sensitivity of 90% and significance level of 0.01. Specifically, the standard deviation considered in power analysis is based on a Bernoulli distribution for each cell. We assumed that the probability of a heterozygous genotype call is the same across all SNVs and each element in Bernoulli distribution for a given cell has a probability of being called as heterozygous based on a conservative approximation of overall rate of heterozygosity in *PIK3CA*^WT^ cells (~80%). For power analysis, we included a further simplifying assumption that all SNPs have a genotype and there are no missing values. Finally, Fig. 2d shows required number of cells for the aforementioned power given a certain number of SNVs in each bin and an expected reduction in proportion of heterozygous calls for a given SNV of either 0.3 (thus, a representative variant cell would be heterozygous in 50% of SNPs for a given bin versus 80% for a representative wild-type cell) or 0.2.

For statistical analyses differentiating distribution of genotypes in cells with and without an activating variant in *PIK3CA* (Fig. 3a), we make two critical assumptions consistent with approach to power analyses.

1. Each cell is an independent Bernoulli distribution with each SNP having a likelihood of being called as heterozygous = Prop_het_
2. Missing values are not explicitly considered. Thus, if in a bin of 6 SNPs for cell A, there are three heterozygous calls, two homozygous calls and 1 missing value, the proportion heterozygous for cell A is 3/5 = 0.6.

After generating a cell level proportion of heterozygous calls across a given predefined region (4 SNPs or chromosomal arms), we perform an unpaired, one-sided, ranked-sum Wilcoxon test comparing the distribution of Prop_het_ between cells with and without an activating variant in *PIK3CA*. Importantly, we only consider cells heterozygous for the activating variant in *PIK3CA*, since homozygous calls may be driven by allelic dropout, technical artifact or loss of portions of chromosome 3, all confounders for our statistical approach, which may ablate our ability to identify somatic LOH in *PDCD10*, which lies on chromosome 3 as well. We then performed Bonferonni correction on p-values with n determined by number of tests given bin size (either set of SNPs or full chromosome arms).

Importantly, there are some SNPs in Fig. 3a within regions of identified somatic LOH that do not diverge noticeably between cell populations. We manually investigated all variants within regions of LOH with Prop_het_ > 0.8 in *PIK3CA*^GoF^ cells. We find that such variants either overlap with an identified indel, are at loci heterozygous for two alternative alleles or are within 10 base pairs of an indel (Supplementary Data 4). Our statistical framework is robust enough to identify somatic LOH without additional filtration and we elected to bypass further curation, since in some instances confounding variants may be present and not identified.

### Co-enrichment of PIK3CA variants and CCM or MAP3K3 variants

We additionally assessed the co-enrichment of variants in the same cells for Figs. 2a, 3c, d and Supplementary Fig. 3b as previously described by Ren and Snellings et al.^19^. Briefly, depth >20 at all loci, alternate allele count >10 and alternate allele frequency >0.1, while *p* values were determined by a two-tailed chi-squared test. We considered the number of double (or triple) mutants compared with the expected number of double (or triple) mutants given the null hypothesis the two variants exist in separate clonal populations at a proportion of 2 * variant allele frequency. The expected number of droplets with two (or more) nuclei was determined by approximating a Poisson distribution and we report the Poisson estimated *p*-values for all tests. We consider homozygous calls identically and can use the rate of homozygous calls as an approximation for variant allele frequency.

For Fig. 3b, we must estimate the enrichment of homozygosity alternately, given the germline deletion spans multiple exons. In order to assess the enrichment of homozygosity, we create more stringent criteria for high confidence variants within *CCM2*. Specifically, variants must have depth >50 and each selected cell must have >50% genotypes present. We then developed a simple statistical test to determine if the proportion of missing genotypes across all SNPs within *CCM2* was heavily enriched in *PIK3CA*^GoF^ cells. We considered a binomial distribution where each cell either had all 5 SNPs with missing genotypes (1) or any of the 5 SNPs was not missing (0). We then used a binomial test to determine p-value. Figure 3b shows randomly selected SNPs outside of *CCM2* for visualization alongside SNPs within Exons 2–10.

### Haplotype bias within regions of somatic LOH

For Supplementary Fig. 4 all SNPs within the region called as somatic LOH were considered as long as they had at least 5 variant cells called as homozygous. Then, we plotted the ratio of max(homozygous_ref_, homozygous_alt_) / total homozygous calls for cells either positive or negative for an activating variant in *PIK3CA*.

### MoChA analysis

To determine whether or not there was evidence of allelic imbalance in 100X WGS data of CCMs 5004 and 5078, we first took all SNPs generated by Novogene and filtered out variants with minor allele count <20. We then only considered variants found in dbSNP and phased variants with ShapeIT4 with reference panels from the 1000 Genomes Project (1kGP). We then imported phased genotypes into the original VCF and added GC content estimations using mochatools, an add-on to bcftools included in MoChA software (software.broadinstitute.org/software/mocha/). We then excluded variants in regions known to be challenging to map using the combined file of all difficult regions provided by genome in a bottle (https://github.com/genome-in-a-bottle/genome-stratifications/tree/master/GRCh38).

Finally, we ran MoChA with LRR and LRR-BAF model turned off, since we were using WGS data (LRR-weight 0.0 and bdev-LRR-BAF) and minimum distance between variants of 400. Plots were generated using MoChA’s included plotting function with WGS parameter selected (Fig. 4b). MoChA calls of allelic imbalance and approximated percentage of cells are included in Supplementary Data 1.

### Reporting summary

Further information on research design is available in the Nature Portfolio Reporting Summary linked to this article.

### Supplementary information

Supplementary Information

Peer Review File

Reporting Summary

Description of Additional Supplementary Files

Supplementary Data 1

Supplementary Data 2

Supplementary Data 3

Supplementary Data 4

Supplementary Data 5

Supplementary Data 6

### Source data

Source Data

## Data Availability

The unprocessed sequencing data generated for this study have been deposited in the GSA database under accession code HRA004839. The processed data generated in this study are provided in Supplementary Data and Source Data file. Both reference genomes hg38 [https://www.ncbi.nlm.nih.gov/datasets/genome/GCF_000001405.40/] and hg19 [https://www.ncbi.nlm.nih.gov/datasets/genome/GCF_000001405.25/] were used in this study and are publicly available. Source Data are provided as a Source Data file. Source data are provided with this paper.

## Code availability

snDNA-sequencing analysis was implemented using Mission Bio’s Mosaic software, which is available on github [https://github.com/MissionBio/mosaic]. MoChA software is available through Broad Institute [https://software.broadinstitute.org/software/mocha/].
