# Supplementary material for: Single-nucleus DNA sequencing reveals hidden somatic loss-of-heterozygosity in Cerebral Cavernous Malformations
Source: Nat Commun. 2023 Nov 2;14:7009. doi: 10.1038/s41467-023-42908-w (PMC10622526; doi:10.1038/s41467-023-42908-w)
Supplement: Supplementary file 1 — Supplementary Information [file 41467_2023_42908_MOESM1_ESM.pdf]

# Supplementary Information
# (Supp. Figures)

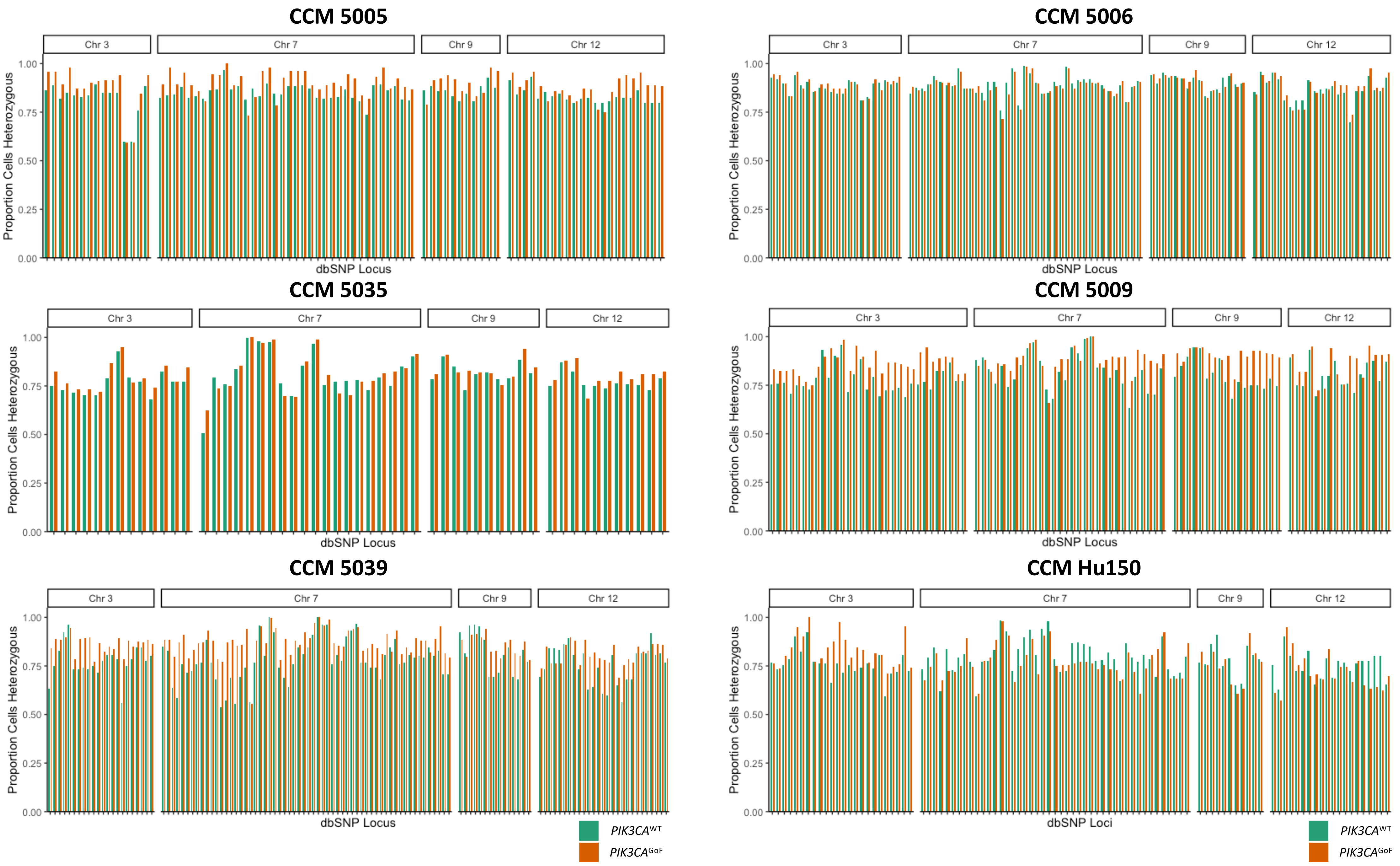

CCM 5005
CCM 5006
CCM 5035
CCM 5009
CCM 5039
CCM Hu150
Chr 3
Chr 7
Chr 9
Chr 12
Proportion Cells Heterozygous
1.00
0.75
0.50
0.25
0.00
dbSNP Locus
dbSNP Loci
PIK3CA^WT
PIK3CA^GoF

Supplementary figure 1: No evidence of somatic LOH in 6 CCM lesions. All graphs show the proportion of cells heterozygous for a given SNP for cells with (orange) and without (teal) an activating variant in *PIK3CA*. No set of 4 SNPs in a given bin or chromosome arm shows evidence of significant (FDR-corrected $p<.05$) reduction in heterozygosity. Exact chromosomal location and rsids for all loci in graph are reported in Supplementary Data 5. Underlying data for bar charts in source data.

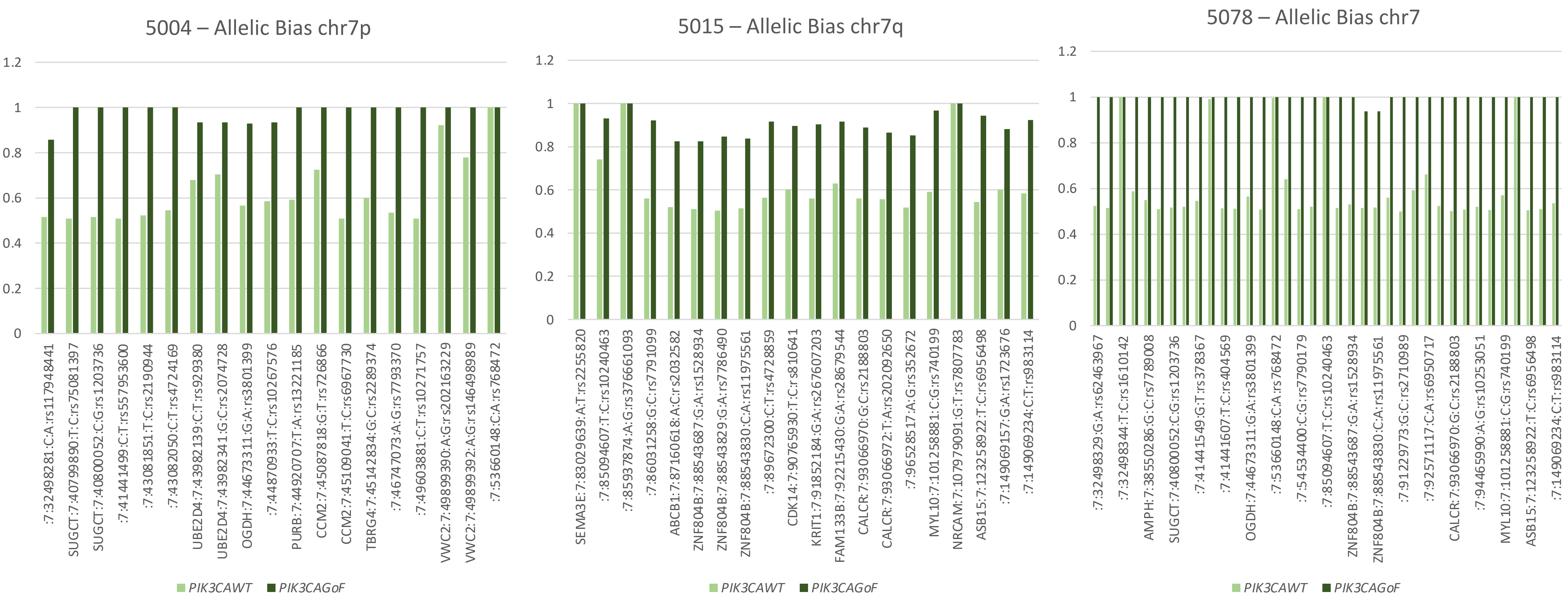


Supplementary figure 2: Allelic bias in regions of somatic LOH. Within the chromosomal arms with LOH identified in figure 3, we plotted the proportion of homozygous calls pertaining to the predominant allele in cells with (dark green) and without (light green) an activating variant in *PIK3CA*. A score of 0.5 is suggestive of random allelic dropout, with each parental allele called as homozygous in equal proportions, whereas a score of 1 means all homozygous calls at a given SNP are for a single parental allele. Underlying data for bar charts included in source data for figure 2.

a

| Sample ID | Inheritance | PIK3CA variant | Lesion Variant 1 | Lesion Variant 2 |
|---|---|---|---|---|
| **5004** | Sporadic | Yes (1%) | S:*CCM2* | LOH (chr7p) |
| **5005** | Sporadic | Yes (2%) | ? | ? |
| **5006** | Sporadic | Yes (8%) | S:*MAP3K3* | |
| **5009** | Sporadic | Yes (<1%) | S:*KRIT1* | S:*KRIT1* |
| **5015** | Familial | Yes (4.2%) | G:*KRIT1* | LOH (chr7q) |
| **5035** | Sporadic | Yes (4.2%) | ? | ? |
| **5039** | Familial | Yes (9%) | G:*KRIT1* | S:*KRIT1* |
| **5078** | Familial | Yes (1%) | G:*CCM2* | LOH (chr7) |
| **Hu150** | Sporadic | Yes (11%) | ? | ? |

b

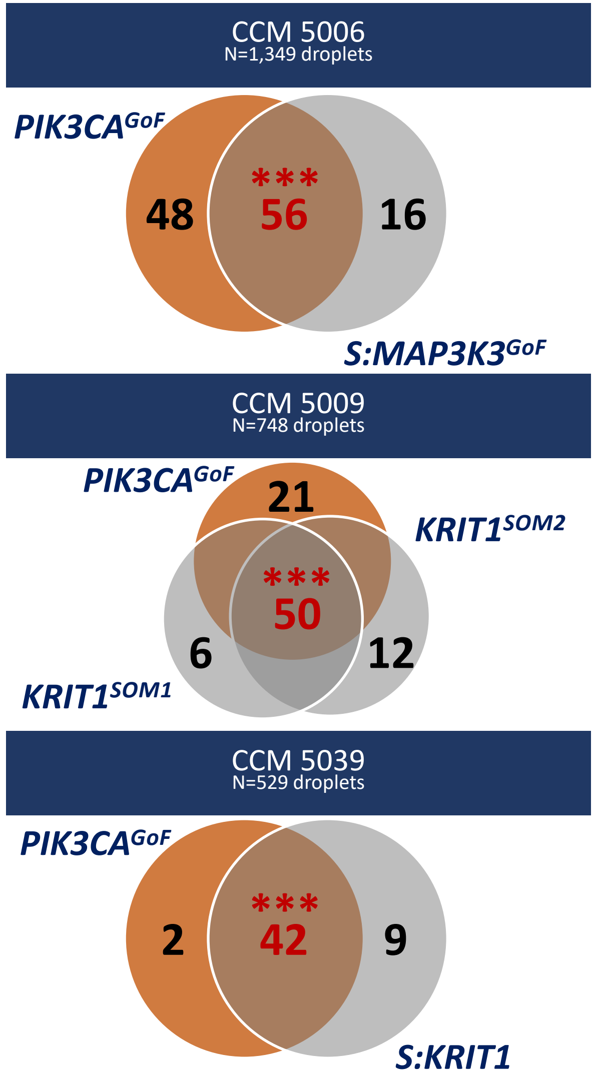


Supplementary figure 3: Summary of genetic diagnoses in CCMs. a) We identified somatic LOH or previously unidentified variants in 6/9 lesions (in gray). b) The identified variants are enriched in cells with activating variants in *PIK3CA* for CCMs 5006, 5009 and 5039. S: Somatic, G: Germline. All variants are specifically reported in Supplementary Data 6. *** p<1e-4. * p < .01. Exact p-values and underlying data for (b) are available in source data, including source data for figures 2a and 3c,d

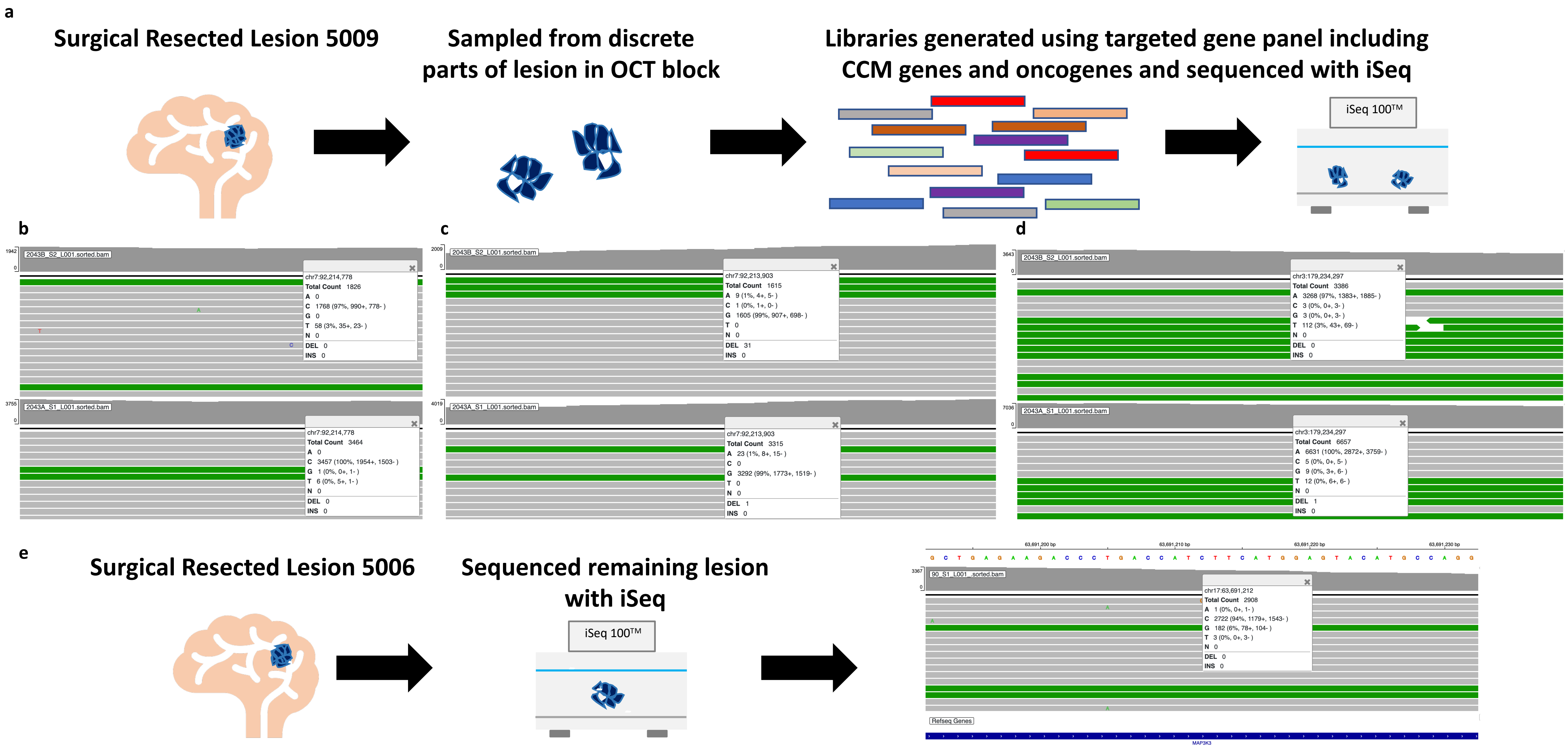
a
Surgical Resected Lesion 5009
Sampled from discrete parts of lesion in OCT block
Libraries generated using targeted gene panel including CCM genes and oncogenes and sequenced with iSeq
iSeq 100TM
b
2043B_S2_L001.sorted.bam
chr7:92,214,778
Total Count 1826
A 0
C 1768 (97%, 990+, 778- )
G 0
T 58 (3%, 35+, 23- )
N 0
DEL 0
INS 0
2043A_S1_L001.sorted.bam
chr7:92,214,778
Total Count 3464
A 0
C 3457 (100%, 1954+, 1503- )
G 1 (0%, 0+, 1- )
T 6 (0%, 5+, 1- )
N 0
DEL 0
INS 0
c
2043B_S2_L001.sorted.bam
chr7:92,213,903
Total Count 1615
A 9 (1%, 4+, 5- )
C 1 (0%, 1+, 0- )
G 1605 (99%, 907+, 698- )
T 0
N 0
DEL 31
INS 0
2043A_S1_L001.sorted.bam
chr7:92,213,903
Total Count 3315
A 23 (1%, 8+, 15- )
C 0
G 3292 (99%, 1773+, 1519- )
T 0
N 0
DEL 1
INS 0
d
2043B_S2_L001.sorted.bam
chr3:179,234,297
Total Count 3386
A 3268 (97%, 1383+, 1885- )
C 3 (0%, 0+, 3- )
G 3 (0%, 0+, 3- )
T 112 (3%, 43+, 69- )
N 0
DEL 0
INS 0
2043A_S1_L001.sorted.bam
chr3:179,234,297
Total Count 6657
A 6631 (100%, 2872+, 3759- )
C 5 (0%, 0+, 5- )
G 9 (0%, 3+, 6- )
T 12 (0%, 6+, 6- )
N 0
DEL 1
INS 0
e
Surgical Resected Lesion 5006
Sequenced remaining lesion with iSeq
iSeq 100TM
63,691,200 bp
63,691,210 bp
63,691,220 bp
63,691,230 bp
90_S1_L001_.sorted.bam
chr17:63,691,212
Total Count 2908
A 1 (0%, 0+, 1- )
C 2722 (94%, 1179+, 1543- )
G 182 (6%, 78+, 104- )
T 3 (0%, 0+, 3- )
N 0
DEL 0
INS 0
Refseq Genes
MAP3K3

Supplementary figure 4: Genetic heterogeneity in CCM 5009. a) Schematic showing targeted bulk DNA-sequencing of two sections of resected lesion CCM 5009. All three variants from CCM lesion 5009 that were identified using snDNA-sequencing (b) hg38 chr7:92214778:C/T, (c) hg38 chr7:92213903:GA/A & (d) hg38 chr3:179234297:A/T) were similarly identified in ~1.8 - 3.3% of reads in one section of lesion 5009 (2043B, (b),(c),(d) top). However, all three variant sequences were identified in less than 0.2% of reads in a second subsection of the same lesion (2043B, (b),(c),(d) bottom) processed, sequenced and analyzed simultaneously, suggesting heterogeneity within the lesion can confound identification of pathogenic variants. e) For CCM 5006, we validated the *MAP3K3* variant hg38 chr17:63691212:C>G with similar strategy, using same panel and targeted bulk DNA-sequencing on one alternative section of the lesion.
