# Supplementary material for: Single-nucleus DNA sequencing reveals hidden somatic loss-of-heterozygosity in Cerebral Cavernous Malformations
Source: Nat Commun. 2023 Nov 2;14:7009. doi: 10.1038/s41467-023-42908-w (PMC10622526; doi:10.1038/s41467-023-42908-w)
Supplement: Supplementary file 3 — Reporting Summary [file 41467_2023_42908_MOESM3_ESM.pdf]

nature portfolio

Corresponding author(s): Andrew K. Ressler, Douglas A. Marchuk

Last updated by author(s): Oct 20, 2023

Reporting Summary

Nature Portfolio wishes to improve the reproducibility of the work that we publish. This form provides structure for consistency and transparency in reporting. For further information on Nature Portfolio policies, see our Editorial Policies and the Editorial Policy Checklist.

Statistics

For all statistical analyses, confirm that the following items are present in the figure legend, table legend, main text, or Methods section.

n/a	Confirmed	
☐	☒	The exact sample size (*n*) for each experimental group/condition, given as a discrete number and unit of measurement
☐	☒	A statement on whether measurements were taken from distinct samples or whether the same sample was measured repeatedly
☐	☒	The statistical test(s) used AND whether they are one- or two-sided<br>*Only common tests should be described solely by name; describe more complex techniques in the Methods section.*
☒	☐	A description of all covariates tested
☐	☒	A description of any assumptions or corrections, such as tests of normality and adjustment for multiple comparisons
☐	☒	A full description of the statistical parameters including central tendency (e.g. means) or other basic estimates (e.g. regression coefficient) AND variation (e.g. standard deviation) or associated estimates of uncertainty (e.g. confidence intervals)
☐	☒	For null hypothesis testing, the test statistic (e.g. *F*, *t*, *r*) with confidence intervals, effect sizes, degrees of freedom and *P* value noted<br>*Give P values as exact values whenever suitable.*
☒	☐	For Bayesian analysis, information on the choice of priors and Markov chain Monte Carlo settings
☒	☐	For hierarchical and complex designs, identification of the appropriate level for tests and full reporting of outcomes
☒	☐	Estimates of effect sizes (e.g. Cohen's *d*, Pearson's *r*), indicating how they were calculated

Our web collection on statistics for biologists contains articles on many of the points above.

Software and code

Policy information about availability of computer code

Data collection	Data was collected from Mission Bio's Tapestri platform, Illumina's iSeq sequencing machine and Novogene for whole genome sequencing.
Data analysis	GATK best practices were used for sequencing analysis and all software is publicly available. MoChA was used and is available through Broad institute. Mission Bio's mosaic software was used for snDNA-sequencing analysis and is similarly available online.

For manuscripts utilizing custom algorithms or software that are central to the research but not yet described in published literature, software must be made available to editors and reviewers. We strongly encourage code deposition in a community repository (e.g. GitHub). See the Nature Portfolio guidelines for submitting code & software for further information.

Data

Policy information about availability of data

All manuscripts must include a data availability statement. This statement should provide the following information, where applicable:

- Accession codes, unique identifiers, or web links for publicly available datasets
- A description of any restrictions on data availability
- For clinical datasets or third party data, please ensure that the statement adheres to our policy

The unprocessed sequencing data generated for this study have been deposited in the GSA database under accession code HRA004839. The processed data generated in this study are provided in Supplementary Tables and Source Data file. Both reference genomes hg38 (https://www.ncbi.nlm.nih.gov/datasets/genome/GCF_000001405.40/) and hg19 (https://www.ncbi.nlm.nih.gov/datasets/genome/GCF_000001405.25/) were used in this study and are publicly available.

Research involving human participants, their data, or biological material

Policy information about studies with human participants or human data. See also policy information about sex, gender (identity/presentation), and sexual orientation and race, ethnicity and racism.

Reporting on sex and gender	We only use de-identified data, but did perform whole genome sequencing, thus gender is known for patients with CCM lesions 5004 and 5078.
Reporting on race, ethnicity, or other socially relevant groupings	De-identified data was used, not applicable.
Population characteristics	We use De-identified data, not applicable.
Recruitment	No patients were recruited for this work
Ethics oversight	Our research complies with all relevant ethical regulations, including the Declaration of Helsinki and has been approved by the Institutional Review Boards of University of Chicago, Duke University and the Alliance to Cure Cavernous Malformations.

Note that full information on the approval of the study protocol must also be provided in the manuscript.

Field-specific reporting

Please select the one below that is the best fit for your research. If you are not sure, read the appropriate sections before making your selection.

[x] Life sciences   [ ] Behavioural & social sciences   [ ] Ecological, evolutionary & environmental sciences

For a reference copy of the document with all sections, see nature.com/documents/nr-reporting-summary-flat.pdf

Life sciences study design

All studies must disclose on these points even when the disclosure is negative.

Sample size	There were 10 lesions considered each from different patients. This sample size was chosen based on existence of sufficient tissue from CCMs previously sequenced that had an activating variant in PIK3CA and 0 or 1 identified loss-of-function variants in a CCM gene. The sample size was strictly determined by availability of tissue and no statistical method was chosen to determine the sample size in advance.
Data exclusions	No data was excluded
Replication	We sequence resected lesions from patients and there is limited material and intralesional heterogeneity, so repeating a snDNA-sequencing on the same sample was not attempted. However, we do validate our findings using an orthogonal method (either targeted bulk dna-sequencing or 100X whole genome sequencing) in 3/4 samples where we attempted to do so. The 4th sample is not a failure of replication, as our approach was developed due to limitations of existing methods to identify allelic imbalance in a small number of cells and we believe this shows the benefits of our method.
Randomization	Not applicable as all lesions sequenced were resected from patients and pathogenic. Samples are primary brain tissue from patients following surgery. We rarely have access to surgically resected brain tissue without a diagnosis and when studying the genetics of CCMs, it is common to only consider the genetics of the lesions themselves. On occasion, we have access to control brain tissue from same patients, but that was not the case for any of the samples we considered, so we cannot randomize between healthy tissue and lesional tissue, as we only sequenced lesional tissue.
Blinding	Blinding is not applicable for our study as all lesions sequenced were resected from patients and pathogenic. Same as with randomization, we are sequencing resected brain tissue from patients with aggressive CCMs and there is no matching non-lesional tissue from these brains. Further, we are only studying the genetic underpinnings of Cerebral Cavernous Malformations and only CCM patient tissue was considered for this work. Since all samples share the same rare disease, blinding is not applicable.

Reporting for specific materials, systems and methods

We require information from authors about some types of materials, experimental systems and methods used in many studies. Here, indicate whether each material, system or method listed is relevant to your study. If you are not sure if a list item applies to your research, read the appropriate section before selecting a response.

Materials & experimental systems

n/a	Involved in the study
☒	☐ Antibodies
☒	☐ Eukaryotic cell lines
☒	☐ Palaeontology and archaeology
☒	☐ Animals and other organisms
☒	☐ Clinical data
☒	☐ Dual use research of concern
☒	☐ Plants

Methods

n/a	Involved in the study
☒	☐ ChIP-seq
☒	☐ Flow cytometry
☒	☐ MRI-based neuroimaging

Plants

Seed stocks

no plants were used

Novel plant genotypes

no plants were used

Authentication

no plants were used
