# Supplementary material for: Single-nucleus DNA sequencing reveals hidden somatic loss-of-heterozygosity in Cerebral Cavernous Malformations
Source: Nat Commun. 2023 Nov 2;14:7009. doi: 10.1038/s41467-023-42908-w (PMC10622526; doi:10.1038/s41467-023-42908-w)
Supplement: Supplementary file 4 — Description of Additional Supplementary Files [file 41467_2023_42908_MOESM4_ESM.pdf]

Description of Additional Supplementary Materials:

Supplementary Data 1: MoChA Calls for CCMs 5004 & 5078

Supplementary Data 2: Average depth at all loci included in figure 3 are shown, with average depth across all loci, or across all loci within region of somatic LOH, listed in the first column. Depths shown for cells wild-type for PIK3CA or heterozygous for the identified activated variant in PIK3CA

Supplementary Data 3: Amplicons included in snDNA-sequencing. All loci pertain to hg19

Supplementary Data 4: Graph Outliers, PIK3CA GoF Cells Heterozygous > 0.8 at loci. All loci pertain to hg19

Supplementary Data 5: Data used to generate graphs in figures 2,3 and supplementary figure 1. Chromosomal locations pertain to hg19 and values in WT and GOF columns represent proportion of cells called as heterozygous in PIK3CA wild-type and gain-of-function cells respectively

Supplementary Data 6: All SNPs and Indels included in manuscript
