# Supplementary material for: Single-nucleus DNA sequencing reveals hidden somatic loss-of-heterozygosity in Cerebral Cavernous Malformations
Source: Nat Commun. 2023 Nov 2;14:7009. doi: 10.1038/s41467-023-42908-w (PMC10622526; doi:10.1038/s41467-023-42908-w)
Supplement: Supplementary file 2 — Peer Review File [file 41467_2023_42908_MOESM2_ESM.pdf]

nature portfolio

# Peer Review File

Single-nucleus DNA sequencing reveals hidden somatic loss-of-heterozygosity in Cerebral Cavernous Malformations.

**Open Access** This file is licensed under a Creative Commons Attribution 4.0 International License, which permits use, sharing, adaptation, distribution and reproduction in any medium or format, as long as you give appropriate credit to the original author(s) and the source, provide a link to the Creative Commons license, and indicate if changes were made. In the cases where the authors are anonymous, such as is the case for the reports of anonymous peer reviewers, author attribution should be to 'Anonymous Referee' followed by a clear attribution to the source work. The images or other third party material in this file are included in the article's Creative Commons license, unless indicated otherwise in a credit line to the material. If material is not included in the article's Creative Commons license and your intended use is not permitted by statutory regulation or exceeds the permitted use, you will need to obtain permission directly from the copyright holder. To view a copy of this license, visit http://creativecommons.org/licenses/by/4.0/.

Reviewer #1 (Remarks to the Author):

Andrew K. Ressler and colleagues present very interesting data on single-nucleus sequencing of multiple human CCM lesions. They have identified somatic loss-of-heterozygosity in three samples and uncovered previously hidden somatic mutations in three others. The manuscript is well written and easy to follow.

The manuscript seems very promising. The presented results are highly relevant for the CCM community and also demonstrate the power of single nucleus sequencing, which is also relevant for other diseases.

However, I think there are some points that should be addressed to increase the readability and clarity of the manuscript:

- The term "complete genetic diagnosis" may be appropriate in a research context, but can be misleading in a more general context. Since only the smaller proportion of CCM patients undergoes surgery, identifying somatic variants is not an option for many CCM patients. To avoid confusion, a clearer differentiation should be made between a research approach and a diagnostic approach that still focuses on germline testing.

- The authors might reconsider the use of the term "homozygous" (e.g., in Figure 3) as homozygosity is defined as the "presence of two identical alleles at a particular gene locus". If there is a SNP on one allele and a large deletion on the other, one should probably not call it homozygosity for the SNP. Perhaps "homozygous call", "apparently homozygous" or something similar might be more appropriate. The specification might be added to the Figure legend.

- The known germline variants and the newly identified somatic SNV/indel variants should be named in an extended data table in a HGVS-compliant manner.

- The target region of the amplicon panel used for single nucleus sequencing should be specified. Were all exons of the genes, only the coding exons or only hot spot regions covered?

- The resolution of the heterozygosity plots (Fig. 2c, 3a, Extended data figure 1) is quite low in both the PDF and the Word file. The text information is barely readable.

- The figure legends for extended data figures 2 and 3 do not match the figures (mixed up?).

- Since different reference genomes were used in the sequencing approaches, it would be helpful to refer to hg19 or hg38 when giving chromosomal coordinates.

- Some abbreviations in the manuscript needs to be explained (e.g., "OCT").

- Line 353: "Wilcoxon text" -> "Wilcoxon test"; Line 513: "homozygous (CCM2+/-)" -> "homozygous (CCM2-/-)"; Lines 47-48: "CCM signaling complex (CSC), comprised of three CCM genes KRIT1, CCM2 and PDCD10" -> "CCM signaling complex (CSC), comprised of three CCM proteins KRIT1, CCM2 and PDCD10"?

- "Author contribution", "Competing interest" and "Data availability" statements should be added in the manuscript file.

Reviewer #2 (Remarks to the Author):

The manuscript by Ressler et al used novel single-nucleus DNA-sequencing (to identify a novel genetic mechanism for cerebral cavernous malformation (CCM) pathogenesis. The study is extremely novel and significant as the genetic mechanisms responsible for CCM development remain largely heterogenous and unknown. This is study is the first to use single-nucleus DNA sequencing in CCM pathogenesis. Although the findings does not lead to immediate improvement

of CCM patient outcomes, but the findings has pioneered steps to fully characterise CCM patient genotypes. This is critical as CCM patients exhibit broad range of symptoms from asymptomatic to severe seizures and strokes. Hence, the findings from this study can improve the diagnosis of CCM patients at genotypic level to provide better evaluation and monitoring for individuals. The authors have drawn very insightful and highly thoughtful conclusions and discussion from their study, demonstrating their in-depth expertise in CCM pathogenesis. Overall, the study is well designed and the manuscript is very well written with very few minor errors.

One major suggestion is whether the authors have investigated other known mutations in PDCD10 (aka CCM3), STKs, CDC42 genes which have been shown to cause CCM lesions in humans or animal models.

Reviewer #3 (Remarks to the Author):

The authors have developed a computational method to identify somatic LOH and reported somatic LOH in a portion of CCM cases as the mechanism to support the "three-hit" mutational hypothesis. Overall, the manuscript is well-written. Yet, there are some major concerns:

1. According to snDNA-seq data of CCM5075 in Figure 2, there are 3 cells carrying PIK3CA H1047R together with CCM2+/+. According to Figure 1b, this patient should have the germline mutation of CCM2. Therefore, the existence of the 3 cells might indicate the potential false positive rate of the technology. Please carefully evaluate the potential impact of this issue on the conclusion.

2. According to snDNA-seq data of CCM5075 in Figure 2, there are 8 cells carrying PIK3CA mutation together with CCM2+/-. Is this an indication that PIK3CA might occur early than CCM2 LOH?

3. Please also list the numbers of PIK3CA_WT cells in Figure 2b (CCM2+/+, CCM2+/-, CCM2-/-). These numbers will provide a better picture for understanding the false positives and false negatives of this technology.

4. The statistic model proposed for LOH should also consider read depth at each position. No such data have been provided throughout the whole manuscript. This is a piece of very important data for evaluating the reliability of the results.

5. The authors claimed complete genetic diagnosis for 6/9 samples. Their results need to be further validated via independent experimental methods.

6. 100x whole genome sequencing analysis needs to be further elaborated to demonstrate the direct evidence of LOH. Only computational inference is kind of weak.

7. Read depth at LOH regions might reflect whether the regions are copy number loss or cnLOH.

There are also some minor comments:

1. Line 106: with regions of significant variation on chromosomes 3,7,9 and 12. Gene names and loci of the significant variation should be stated before introducing the selection of regions.

2. Line 108: the concept of "complete diagnosis" is
not academically rigorous.

3. Line 123: Is "Figure 1b" a typo?

4. Figure legend is too tiny to be recognized in several figures.

5. Figure 4b: what is the indication of LOD 34.20?

**REVIEWER COMMENTS**

Reviewer #1 (Remarks to the Author):

Andrew K. Ressler and colleagues present very interesting data on single-nucleus sequencing of multiple human CCM lesions. They have identified somatic loss-of-heterozygosity in three samples and uncovered previously hidden somatic mutations in three others. The manuscript is well written and easy to follow.

The manuscript seems very promising. The presented results are highly relevant for the CCM community and also demonstrate the power of single nucleus sequencing, which is also relevant for other diseases.

However, I think there are some points that should be addressed to increase the readability and clarity of the manuscript:

- The term "complete genetic diagnosis" may be appropriate in a research context, but can be misleading in a more general context. Since only the smaller proportion of CCM patients undergoes surgery, identifying somatic variants is not an option for many CCM patients. To avoid confusion, a clearer differentiation should be made between a research approach and a diagnostic approach that still focuses on germline testing.

**Thank you. We agree and have substituted the term "complete somatic mutation profile".**

- The authors might reconsider the use of the term "homozygous" (e.g., in Figure 3) as homozygosity is defined as the "presence of two identical alleles at a particular gene locus". If there is a SNP on one allele and a large deletion on the other, one should probably not call it homozygosity for the SNP. Perhaps "homozygous call", "apparently homozygous" or something similar might be more appropriate. The specification might be added to the Figure legend.

**Thank you. We agree and have specified "called as homozygous" or "homozygous calls" throughout the manuscript.**

- The known germline variants and the newly identified somatic SNV/indel variants should be named in an extended data table in a HGVS-compliant manner.

**Thank you. We have added SNV/indel variants in extended data table 6.**

- The target region of the amplicon panel used for single nucleus sequencing should be

specified. Were all exons of the genes, only the coding exons or only hot spot regions covered?

**We are sorry for this omission. We have added a note in methods for targeted DNA-sequencing and single nucleus DNA-sequencing to specify that all exons of the three CCM genes were covered, but only mutation hotspot exons for *PIK3CA* and *MAP3K3* were covered. Additionally, we have added extended data table 3, which includes all the regions covered by the amplicon panel used for single nucleus sequencing, so that readers know exactly which regions were amplified.**

- The resolution of the heterozygosity plots (Fig. 2c, 3a, Extended data figure 1) is quite low in both the PDF and the Word file. The text information is barely readable.

**We agree that the resolution was low. Each column has an rsid associated, but regardless of font changing, including rsids clearly readable was challenging given the the amount of space available in the figure. In order to address this, we have removed the rsids and noted in the figure legends where the underlying rsids with each set of columns can be found (Extended Data Table 5). This file includes all the data points used for the graphs in figures 2c, 3a and Extended data figure 1.**

- The figure legends for extended data figures 2 and 3 do not match the figures (mixed up?).

**Thank you for noticing this mistake. The legends were accidentally swapped and this has been corrected.**

- Since different reference genomes were used in the sequencing approaches, it would be helpful to refer to hg19 or hg38 when giving chromosomal coordinates.

**Thank you for this suggestion. We have added hg19 and hg38 to variants, as appropriate. We have also added all variants with hg38 chromosomal locations to extended data figure 3.**

- Some abbreviations in the manuscript needs to be explained (e.g., “OCT”).

**We agree. We have defined the acronym (Optimal Cutting Temperature) and provided the manufacturer and trademark.**

- Line 353: “Wilcoxon text” -> “Wilcoxon test”; Line 513: “homozygous (CCM2+/-)” -> “homozygous (CCM2-/-)”; Lines 47-48: “CCM signaling complex (CSC), comprised of three CCM genes KRIT1, CCM2 and PDCD10” -> “CCM signaling complex (CSC), comprised of three CCM proteins KRIT1, CCM2 and PDCD10”?

**We apologize for this mistake. The term "proteins" is correct. Thank you. This has been corrected in the manuscript.**

- "Author contribution", "Competing interest" and "Data availability" statements should be added in the manuscript file.

**We have added this information to the manuscript file.**

Reviewer #2 (Remarks to the Author):

The manuscript by Ressler et al used novel single-nucleus DNA-sequencing (to identify a novel genetic mechanism for cerebral cavernous malformation (CCM) pathogenesis. The study is extremely novel and significant as the genetic mechanisms responsible for CCM development remain largely heterogenous and unknown. This is study is the first to use single-nucleus DNA sequencing in CCM pathogenesis. Although the findings does not lead to immediate improvement of CCM patient outcomes, but the findings has pioneered steps to fully characterise CCM patient genotypes. This is critical as CCM patients exhibit broad range of symptoms from asymptomatic to severe seizures and strokes. Hence, the findings from this study can improve the diagnosis of CCM patients at genotypic level to provide better evaluation and monitoring for individuals. The authors have drawn very insightful and highly thoughtful conclusions and discussion from their study, demonstrating their in-depth expertise in CCM pathogenesis. Overall, the study is well designed and the manuscript is very well written with very few minor errors.

One major suggestion is whether the authors have investigated other known mutations in PDCD10 (aka CCM3), STKs, CDC42 genes which have been shown to cause CCM lesions in humans or animal models.

**Thank you for this suggestion. We have added a note to the amplicon panel description in methods that all coding exons (and flanking intron-exon borders) of *PDCD10* were included in the panel. As for other possible CCM genes, we agree that there likely remain additional genes that when mutated, could cause CCMs, including *STKs* and *CDC42*. We continue to augment our probe capture array with new genes for sequencing in order to discover somatic mutations in other genes. However, in the current work, the target capture panel we used was our previously published panel (Ren et al 2021, PMID: 33910229; Snellings et al., 2022, PMID: 35355835). For this panel, all exons of *PDCD10* were covered, but *STK* genes and *CDC42* were notably absent. There are additional genes of interest for us as well, such as *KLF2* and *KLF4* and we, and others, will continue to develop panels that cover genes recently hypothesized as implicated in CCMs. There is a trade-off between number of genes covered and cost and depth of sequencing, so designing the perfect panel remains a**

**challenge, but one we, and presumably others in the field, intend to continue to pursue.**

**In light of this important point, we have added an additional sentence to the end of discussion to drive home the point that other genes have been, and likely will continue to be, implicated in CCM formation and that sequencing the three CCM genes, *PIK3CA* and *MAP3K3* alone may not identify all possible mutations in CCM lesions.**

Reviewer #3 (Remarks to the Author):

The authors have developed a computational method to identify somatic LOH and reported somatic LOH in a portion of CCM cases as the mechanism to support the "three-hit" mutational hypothesis. Overall, the manuscript is well-written. Yet, there are some major concerns:

1. According to snDNA-seq data of CCM5075 in Figure 2, there are 3 cells carrying PIK3CA H1047R together with CCM2+/+. According to Figure 1b, this patient should have the germline mutation of CCM2. Therefore, the existence of the 3 cells might indicate the potential false positive rate of the technology. Please carefully evaluate the potential impact of this issue on the conclusion.

2. According to snDNA-seq data of CCM5075 in Figure 2, there are 8 cells carrying PIK3CA mutation together with CCM2+/-. Is this an indication that PIK3CA might occur early than CCM2 LOH?

3. Please also list the numbers of PIK3CA_WT cells in Figure 2b (CCM2+/+, CCM2+/-, CCM2-/-). These numbers will provide a better picture for understanding the false positives and false negatives of this technology.

**We thank the reviewer for questions 1-3, which are interrelated. To address question 1, our interpretation of the Heterozygous cells and added a row to figure 2b to include the proportion of *PIK*$^{WT}$ cells with each genotype of the somatic CCM2 variant. As we and others have noted, here is significant noise / variability in single nucleus DNA-sequencing, particularly when considering a single locus. We report the existence of 30/41 homozygous calls at the locus of the known somatic variant as confirmatory of complete loss, with those calls being driven by the germline exon 2-10 deletion that includes that locus.**

**To address question 2, we believe the 8 nuclei called as heterozygous are technical artifacts, since the presence of the large germline deletion can only result in WT or Homozygous calls in the absence of UPD of the WT allele. However, as is well**

**known in single cell/nucleus sequencing, there can be significant rates of doublets and allelic dropout in individual oil droplets, which will lead to such artifacts. Thus, this result is not particularly surprising, but we nonetheless discuss this in the manuscript. Critically, we compare populations of PIK3CA$^{WT}$ and PIK3CA$^{GoF}$ cells across several adjacent SNPs for our power analysis (figure 2) and our identified regions of LOH are across entire chromosome arms with > 20 SNPs. We use the single locus analyses only in concert with identified LOH across a larger region to suggest somatic LOH. We believe this approach is robust to the aforementioned technical artifacts.**

**Like the reviewer, we have been interested in the order of acquisition of somatic mutations when more than one gene is mutated. To address the question of whether *PIK3CA* mutations might occur prior to the somatic single base pair deletion, we do note that there are 3 cells that do not show the somatic variant at the *PIK3CA* locus. Previous single nucleus sequencing from our lab (Ren et al 2021, PMID: 33910229), in addition to the single nucleus data from this manuscript, is gradually increasing the sample size for CCMs that are WT or mutant for *PIK3CA* and/or a CCM gene or *MAP3K3.***

**However, as each individual CCM could have a unique mutational order, we feel we cannot determine which mutation came first in individual CCMs based off the sequencing results alone. Hopefully, as more single-nucleus data is available, our lab or others will design and execute an experimental paradigm that allows clear determination of the order sequence of variants. We do have our theories, especially in CCMs that develop adjacent to *PIK3CA*-positive Developmental Venous Anomalies (Snellings et al., 2022, PMID: 35355835), where we have shown that the DVA exhibits the *PIK3CA* somatic mutation and the adjacent CCM exhibits both the same PIK3CA somatic mutation and an additional *MAP3K3* mutation. That result clearly suggested the order of mutational events. However, in this current manuscript, and based off this data alone, we are not able to confidently determine the sequential order of mutations.**

**Regarding question 3, we have made the requested changes in figure 2b.**

4. The statistic model proposed for LOH should also consider read depth at each position. No such data have been provided throughout the whole manuscript. This is a piece of very important data for evaluating the reliability of the results.

**Thank you for this suggestion. We agree that read depth information may be critical in determining whether somatic LOH is copy neutral or copy loss. While we do not dynamically consider read depth, we do employ a read depth threshold of 10, where any locus with less than 10 reads in any nucleus is not genotyped and that locus is not considered in LOH calculation. This threshold value of 10 reads was chosen in consultation with Mission Bio, the manufacturers of the Tapestri platform. Greater**

**than 10 reads per nucleus provided a sufficient number of informative loci for each nucleus, while excluding loci with unreliable genotypes. Per the reviewer's suggestion, we have added a sentence in the Discussion and included data (now extended data table 2) on depth of coverage for samples where we identified somatic LOH.**

**Importantly, depth of coverage is similar between WT and *PIK*$^{GoF}$ cells across all loci, as well as specifically within the region of somatic LOH, with average read depths in the high 40s to low 60s (well above the 10-read threshold recommended by Mission Bio). We feel confident that we have sufficient read depth to support our conclusions of LOH; however, there is significant variability in depth of coverage from cell to cell, so we are unable to fully rule out copy loss by statistical inference alone.**

5. The authors claimed complete genetic diagnosis for 6/9 samples. Their results need to be further validated via independent experimental methods.

**We appreciate this concern and have since re-sequenced another subsection of CCM 5006 and validated the MAP3K3 variant. We have added that validation to extended data figure 4, which now is shown alongside the validation of both newly identified somatic variants of CCM 5009. For validation of somatic LOH, we have used MoChA, a commonly used method with higher reported sensitivity than alternative bulk approaches. We were pleased that we were able to validate somatic LOH in one of the two CCMs (CCM 5078) we tested, especially given that the mutant allele frequencies we were working with in both our samples were below the detection rates claimed by the authors of MoChA. We appreciate and agree with benefits of external validation and are happy to report we were able to sequence another section of CCM 5006 and externally validate an additional sample.**

6. 100x whole genome sequencing analysis needs to be further elaborated to demonstrate the direct evidence of LOH. Only computational inference is kind of weak.

**We note that MoChA is not our computational technique. However, it is one of the few currently established methods to identify allelic imbalance in a small fraction of a population of cells. We absolutely agree there are weaknesses in attempting to identify LOH using bulk sequencing. This was in large part the motivation for developing our single nucleus-based approach, where we can examine the distribution of genotypes in subpopulations of cells at cell-population level resolution. We merely report the MoChA results to compare our novel approach to an established method to identify allelic imbalance in a small population of cells.**

7. Read depth at LOH regions might reflect whether the regions are copy number loss or cnLOH.

**We had corresponded with the creator of MoChA and while he was confident in identifying allelic imbalance in a small population of cells, when the imbalance occurs in less than 5% of the cells, he warned us that read depth becomes an unreliable indicator of copy number, as technical artifacts begin to confound. With higher proportions of cells, depth is a critical variable in determining copy number associated with allelic imbalance using MoChA. Although we agree with the rationale behind this suggestion, we went with a more conservative approach of not making claims on copy number based on depth in our single nucleus data, given the variability in depth from cell to cell.**

There are also some minor comments:

1. Line 106: with regions of significant variation on chromosomes 3,7,9 and 12. Gene names and loci of the significant variation should be stated before introducing the selection of regions.

**Reviewer 1 had a similar suggestion. We have included more information on the amplicon panel and the regions captured in extended data table 3 so that the locations considered are fully transparent. We also wish to point out that other than the CCM 1, 2, and 3 gene exons and the hotspot exons for the PIK3CA and MAP3K3 somatic mutations, the amplicon panel was chosen to cover regions where there was high level of variation in the genome. The Tapestri platform cannot handle coverage of the whole genome, so we focused on the chromosomes that hold the CCM genes, and one other chromosome as control. But the panel coverage was agnostic to other genes, and instead focused on regions of high heterozygosity (SNPs), which often fell <u>between</u> genes.**

2. Line 108: the concept of "complete diagnosis" is not academically rigorous.

**We agree. Reviewer 1 had the same comment and we have revised to "complete mutation profile".**

3. Line 123: Is "Figure 1b" a typo?

**Yes. Thank you. We have corrected this to "Figure 2b" which corresponds to the text.**

4. Figure legend is too tiny to be recognized in several figures.

**Reviewer 1 had the same comment. As stated above, due to space constraints, we have removed rsid from the figures themselves and included them in extended data table 5.**

5. Figure 4b: what is the indication of LOD 34.20?

**The LOD score is Logarithm of the Odds, the prescribed analysis method used in MoChA. A LOD score of 3 corresponds to a 1 in 10^3 (1,000) chance of the expected outcome occurring by chance. With MoChA, LOD score thresholds of >5 and >10 (1 in 10^5 or 1 in 10^10 odds) are commonly used. The reported LOD score of 34.20 in our data is based on the B-allele frequencies and phase concordance. A LOD score of 34.20 is therefore highly significant, well beyond the suggested thresholds, and indicative of allelic imbalance from telomere to telomere of chromosome 7 in a small proportion of cells. We believe this is strong validation of our result for CCM5078 using our snDNA-sequencing approach. In addition to the LOD score itself, what was most notable to us was that the evidence of allelic imbalance matched the entirety of chromosome 7 that we saw in snDNA-sequencing, in a similarly small cell fraction (~1.8% estimated by MoChA) and was inferred from >50,000 heterozygous sites. For this sample, MoChA and our snDNA-sequencing results were in complete concordance.**

Reviewer #1 (Remarks to the Author):

The revised manuscript by Andrew K. Ressler and colleagues sufficiently answers almost all the questions I raised before. However, the new information on the target region of the custom amplicon panel used for single-nucleus DNA sequencing (Extended Data Table 3) raises an important issue that should be addressed: While the first coding exon of an alternative CCM2 transcript seems to be covered (chr7:45067170-45067416), the first coding exon of the CCM2 MANE Select transcript NM_031443.4 (= is used in most CCM2 mutation databases and corresponds to LRG_664t2) was obviously not covered ((hg19) Chr7:45039787-45039962). Several CCM2 mutations (some of them more frequent) are located in this region. As providing a "complete mutation profile" is an essential point of the manuscript, this important coverage gap should be closed.

Additional (minor) point:
- Regardless of the coverage gap mentioned above, the statement "While all exons were covered for KRIT1, CCM2 and PDCD10, hotspot regions ..." (lines 340+341) should be changed to "While all coding exons were covered for KRIT1, CCM2 and PDCD10, hotspot regions ...". If I see it correctly, non-coding exons of the three genes were not covered.

Reviewer #2 (Remarks to the Author):

Authors have satisfactorily addressed all the reviewers comments.

19 September 2023 - Response to Reviewers

We thank all three reviewers for their helpful comments and suggestions throughout this review process. As always, their suggestions have improved our manuscript. Below we respond to the final comments from Reviewer 1. Our response for the major concern requires an explanation of the two different sequencing technologies used in this study. Each was employed for a *different purpose*, and thus, genomic coverage differed based on the requirements of the phase of the study.

Reviewer #1 (Remarks to the Author):

The revised manuscript by Andrew K. Ressler and colleagues sufficiently answers almost all the questions I raised before. However, the new information on the target region of the custom amplicon panel used for single-nucleus DNA sequencing (Extended Data Table 3) raises an important issue that should be addressed: While the first coding exon of an alternative CCM2 transcript seems to be covered (chr7:45067170-45067416), the first coding exon of the CCM2 MANE Select transcript NM_031443.4 (= is used in most CCM2 mutation databases and corresponds to LRG_664t2) was obviously not covered ((hg19) Chr7:45039787-45039962). Several CCM2 mutations (some of them more frequent) are located in this region. As providing a "complete mutation profile" is an essential point of the manuscript, this important coverage gap should be closed.

**Discovery of somatic point mutations in next-generation sequencing**. Discovery of somatic point mutations was performed for all samples using our *custom-designed* Agilent capture panel for vascular malformation genes. *This panel* does indeed include all coding exons for the three CCM genes, *including the more commonly used exon 1 of the CCM2 gene – the exon that concerns the reviewer.* In that exon, no somatic mutations were identified *that passed our pre-assigned filters*. But per the concern of the reviewer, we reassessed the data for each sample by *visibly* inspecting the BAM files covering the commonly used CCM2 gene exon 1. We searched for any somatic mutation that may have been present but was not flagged because it fell below our QC, allele frequency, and other filter thresholds. However, even with this focused analysis, no somatic mutations were identified in this exon. Thus, we validated our original discovery phase of this work; these samples do not harbor a second-hit somatic point mutation in any coding exon of any of the CCM genes.

**Single nucleus sequencing to identify Loss of Heterozygosity (LOH).** Single nucleus sequencing was performed on the Mission Bio "Tapestri" platform. This platform uses microfluidics to isolate single nuclei (or cells) and then multiplex PCR amplification panels for sequencing in individual droplets. It does not use Agilent capture probes. We worked with Mission Bio to design a *custom amplification panel for our study* that would scan the genomic locus for each of the three CCM genes and extend across a broader area on these chromosomes and one other control chromosome. (There is limited primer capacity available for this single cell/nucleus sequencing platform).

Significantly, our snDNA sequencing was not *designed* to discover new somatic point mutations. That goal was already accomplished by the bulk tissue sequencing using the Agilent capture panel. The Tapestri platform was instead designed to *search for LOH*. PCR primer coverage was designed to include as many heterozygous sites along the chosen chromosomes as possible, capturing areas where SNPs mapped that might reveal LOH. Coverage was highest within, across, and immediately flanking the three CCM genes.

The fact that the exon of concern of reviewer 1, or any other CCM2 exon was not included is not relevant to this study, since the panel was designed to search for LOH across the three CCM genes and their respective chromosomes and was not designed to identify new point mutations.

However, we incidentally identified, and reported, additional point mutations that were not identified using the Agilent capture panel. We provide an explanation for one of these findings (CCM 5009, Extended data figure 4), where we show that targeted DNA-sequencing identifies the variants in one subsection of lesion, but not another, showing the challenges of identifying every low frequency somatic variant. We believe these incidental findings importantly show preliminary evidence of intra-lesional heterogeneity and that re-sequencing with the same or alternative strategies may identify variants that slipped through the cracks due to the inherent biological and technical challenges of identifying low frequency point mutations.

**Could we have missed a somatic point mutation**? This is always a possibility, specifically for the three samples that we still have not identified CCM1,2,3 or MAP3K3 variants. Regardless, *the objective of our study was to determine if LOH is a genetic mechanism for second-hit somatic mutation in CCM lesions*, focusing on samples where we did not find a second-hit somatic point mutation. We were able to meet this goal – showing definitive evidence of LOH as a second-hit somatic mutation in CCM tissue for some of these samples.

Additional (minor) point:
- Regardless of the coverage gap mentioned above, the statement "While all exons were covered for KRIT1, CCM2 and PDCD10, hotspot regions ..." (lines 340+341) should be changed to “While all coding exons were covered for KRIT1, CCM2 and PDCD10, hotspot regions ...”. If I see it correctly, non-coding exons of the three genes were not covered.

**Non-Coding Exons (Minor Comment).** The reviewer is correct that non-coding exons were not covered on our Agilent sequencing panel for point mutation discovery. We might have found low allele frequency sequence variants in non-coding exons of any of the CCM or other genes on our panel, but if so, we would not have (easily) been able to evaluate their functional significance. *Per the request of the reviewer, we have altered the text to state that in our discovery phase using bulk DNA sequencing included all coding exons of the CCM genes.*

We hope these final changes and this explanation concerning CCM2 exon 1 is clear, and that our paper is acceptable for publication in Nature Communications.

Thank you.

Reviewer #1 (Remarks to the Author):

The authors have answered all the questions I raised.
